# Interleukin-6 derived from cancer-associated fibroblasts attenuates the p53 response to doxorubicin in prostate cancer cells

**DOI:** 10.1038/s41420-020-0272-5

**Published:** 2020-06-02

**Authors:** Emarndeena H. Cheteh, Victoria Sarne, Sophia Ceder, Julie Bianchi, Martin Augsten, Helene Rundqvist, Lars Egevad, Arne Östman, Klas G. Wiman

**Affiliations:** 1 Department of Oncology-Pathology, Karolinska Institutet, Stockholm, Sweden; 2 Department of Pathobiochemistry and Genetics, Medical University of Vienna, Vienna, Austria; 3 Department of Laboratory Medicine, Karolinska Institutet, Stockholm, Sweden

**Keywords:** Cancer microenvironment, Cancer therapeutic resistance, Tumour-suppressor proteins

## Abstract

Cancer-associated fibroblasts (CAFs) promote tumor growth and progression, and increase drug resistance through several mechanisms. We have investigated the effect of CAFs on the p53 response to doxorubicin in prostate cancer cells. We show that CAFs produce interleukin-6 (IL-6), and that IL-6 attenuates p53 induction and upregulation of the pro-apoptotic p53 target Bax upon treatment with doxorubicin. This is associated with increased levels of MDM2 mRNA, Mdm2 protein bound to p53, and ubiquitinated p53. IL-6 also inhibited doxorubicin-induced cell death. Inhibition of JAK or STAT3 alleviated this effect, indicating that IL-6 attenuates p53 via the JAK/STAT signaling pathway. These results suggest that CAF-derived IL-6 plays an important role in protecting cancer cells from chemotherapy and that inhibition of IL-6 could have significant therapeutic value.

## Introduction

Cancer-associated fibroblasts (CAFs) are an important component of the tumor microenvironment. CAFs secrete various factors and cytokines that stimulate tumor growth, invasion, and metastasis^1–3^. CAFs can affect the efficacy of chemotherapeutic drugs through various mechanisms. As components of the tumor microenvironment, CAFs regulate interstitial fluid pressure and thus affect the transport of drugs from the vasculature to the tumor interstitium^4^. Moreover, CAFs secrete growth factors, cytokines, and chemokines that increase the resistance of cancer cells to chemotherapy^5^. The molecular mechanisms underlying this chemoresistance-modulating effect of CAF-derived secreted proteins remain only partially defined. Factors such as HGF, IL-6, IL-8, and TGF-β, have been suggested to confer chemoresistance through various mechanisms including induction of EMT or cancer stem cell phenotypes^6–8^. However, the effects of CAF-derived growth factors on chemotherapy-induced p53 responses have not been studied in greater detail.

The TP53 gene encodes the tumor suppressor protein p53, a DNA-binding transcription factor that responds to cellular stress, e.g. DNA damage, and induces cell cycle arrest, apoptosis and/or senescence^9^. p53 can also regulate redox homeostasis^10^ and energy metabolism^11^. Activation of p53 by cellular stress leads to transcriptional transactivation of p53 target genes such as p21 that blocks cell cycle progression, and Bax, Puma, and Noxa that promote apoptosis^12^. The p53-regulated gene MDM2 encodes a ubiquitin ligase that targets p53 for proteasomal degradation, thereby forming a negative feed-back loop that terminates the p53 response to stress^13,14^. p53 is important for the cellular response to DNA-damaging chemotherapeutic drugs, including doxorubicin, 5-fluorouracil, and cisplatin^15–17^. The TP53 gene is mutated in a large fraction of human tumors^18^, in most cases by missense point mutations that disrupt p53’s specific DNA-binding activity^19^. Accordingly, a number of studies have demonstrated that TP53 mutation is associated with increased resistance to chemotherapy and poor prognosis^20,21^. A recent study did not detect any statistically significant survival difference between TP53 wild type and mutant tumors but identified a mutant TP53 RNA expression signature that correlates with survival^22^.

The cytokine IL-6 has been implicated in regulation of tumor cell proliferation and survival, migration, invasion, angiogenesis, and chemotherapy resistance^23^. Enhanced IL-6 levels in tumor tissues or serum are associated with aggressive tumor phenotypes and poor survival in different types of cancer, including prostate cancer^24–27^. IL-6 activates the signal transducer and activator of transcription 3 (STAT3) in both a paracrine and an autocrine manner^28^. The IL-6 receptor is composed of two subunits, IL-6R and signal-transducing protein gp130. Binding of IL-6 to IL-6R leads to gp130-dimerization and activation of JAK kinases. This induces STAT3 dimerization and translocation to the nucleus, where STAT3 dimers activate cancer-related genes including Myc, Bcl-2, and Survivin^28,29^. Constitutive STAT3 phosphorylation and activation is common in tumors and tumor cell lines of various origin^28,30^, indicating a strong link between STAT3 activation and cancer. Clinical trials with inhibitors of the IL-6/JAK/STAT3 signaling pathway are ongoing in several types of tumors^28^.

Previous studies have indicated that p53 expression can be modulated by IL-6 and/or STAT3 at different levels, including TP53 gene transcription and p53 protein degradation^31–33^. We have recently found that CAFs and CAF-conditioned medium attenuate induction of p53 and cell death in doxorubicin-treated prostate cancer cells via secretion of thiol-containing molecules^34^. Given the reported ability of IL-6 to modulate p53 expression, we asked if our prostate CAFs produce cytokines such as IL-6 and if such cytokines might contribute to the effect of CAFs on the p53 response in LNCaP cells that carry wild type p53. Here we show that IL-6 attenuates p53 accumulation and induction of the pro-apoptotic p53 target Bax in doxorubicin-treated LNCaP cells, and inhibits doxorubicin-induced cell death. Inhibitors of JAK or STAT3 abrogate this effect, indicating that IL-6 attenuates p53 via the JAK/STAT signaling pathway. Thus, our results suggest that CAF-derived IL-6 can contribute to protecting cancer cells from chemotherapy and that IL-6 could be a relevant therapeutic target.

## Results

### CAFs secrete cytokines and growth factors

**Fig. 1:**
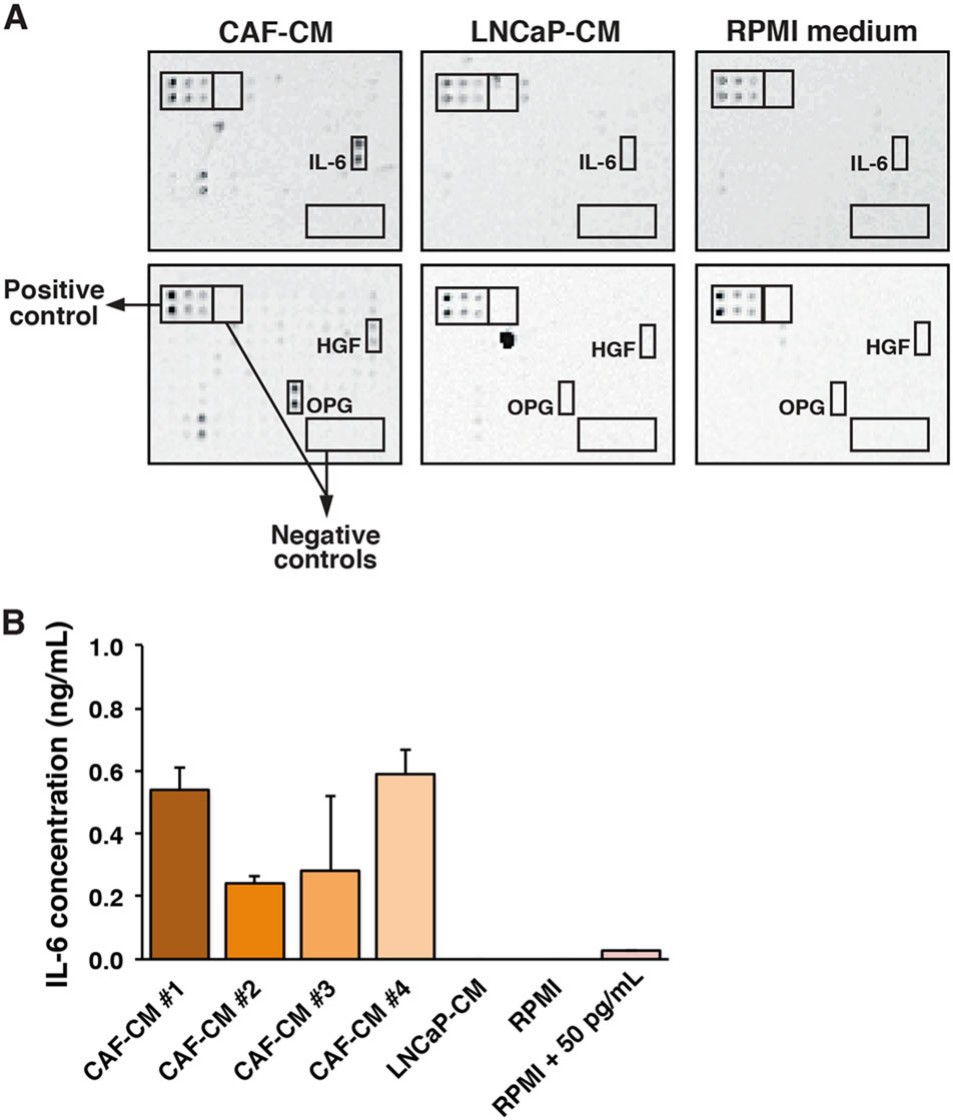
Cytokines and growth factors in CAF-conditioned medium. **a** Cytokine array showing presence of IL-6, HGF, and OPG in conditioned media from CAFs or LNCaP cells or fresh RPMI medium. The array included biotinylated protein as positive control and BSA as negative control. **b** Concentration of IL-6 in conditioned media from CAFs of different prostate cancer patients (#1–4) or LNCaP cells or fresh RPMI medium as assessed by ELISA (*N* ≥ 2). 50 pg/mL IL-6 was added to RPMI medium as positive control.

We first examined the production of soluble factors and cytokines by CAFs using a cytokine array. As shown in Fig. 1a, prostate CAFs secrete several soluble factors, including hepatocyte growth factor (HGF), interleukin-6 (IL-6), and osteoprotegerin (OPG). None of these factors were detected in conditioned medium from the prostate cancer cell line LNCaP or fresh RPMI cell culture medium. We further assessed the levels of IL-6 in CAF-conditioned media from different prostate cancer patients by ELISA and detected IL-6 at concentrations between 0.25 and 0.60 ng/mL in the CAF-conditioned media, while LNCaP did not produce any detectable IL-6 (Fig. 1b).

### IL-6 attenuates the p53 response in prostate cancer cells

**Fig. 2:**
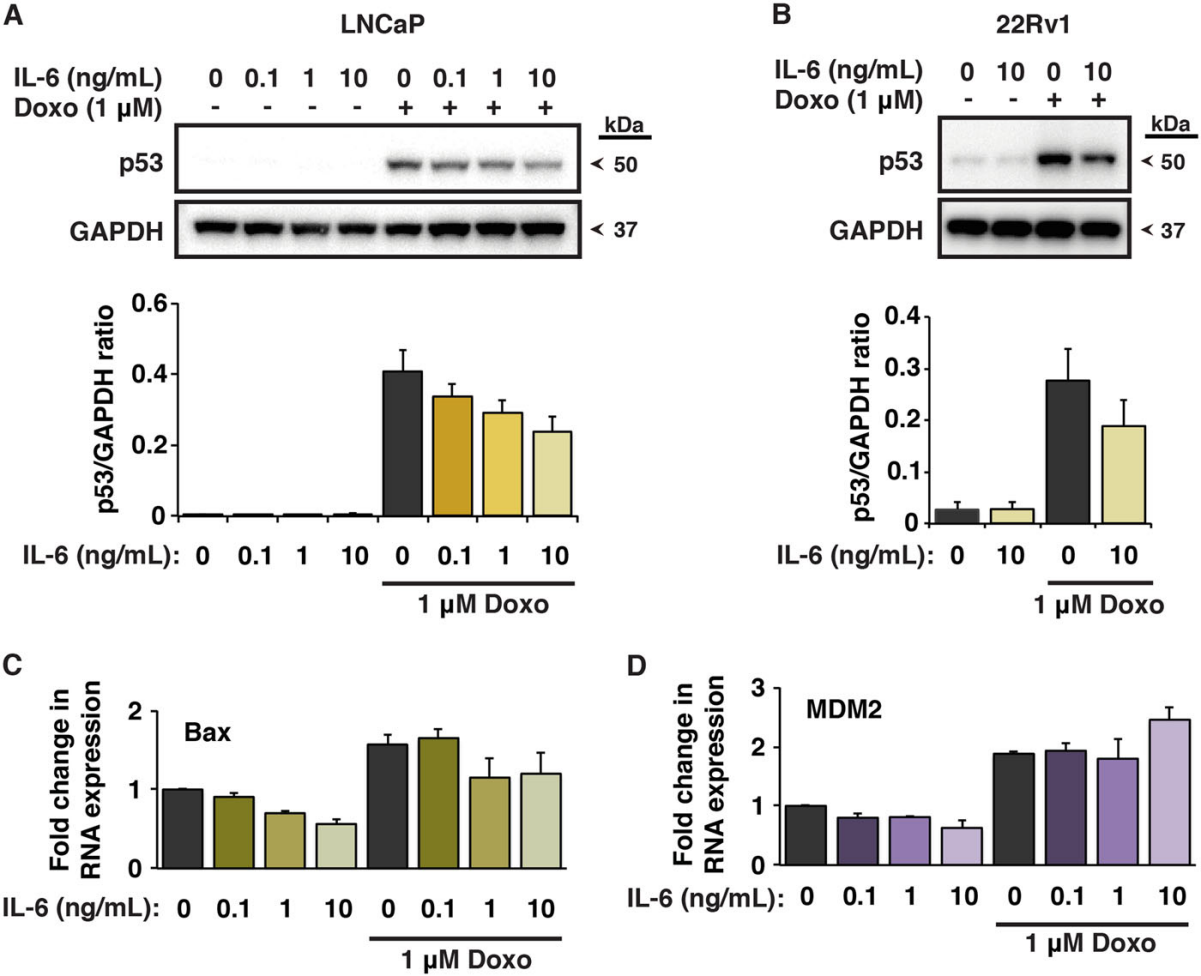
IL-6 attenuates the p53 response in prostate cancer cells. **a** Upper panel: p53 levels in LNCaP cells after treatment with 1 μM doxorubicin for 8 h in the presence or absence of IL-6, as shown by western blotting. Cells were pre-treated with IL-6 (0.1, 1 or 10 ng/mL) for 16 h. The blot was cut into two pieces and probed with anti-p53 DO-1 and anti-GAPDH antibody separately. Lower panel: quantification of p53 levels with or without doxorubicin treatment in the presence of IL-6 (S.E.M. is indicated by bars; *N* = 5). **b** Upper panel: p53 levels in 22Rv1 cells after treatment with 1 μM doxorubicin for 8 h in the presence or absence of 10 ng/mL IL-6, as shown by western blotting. Cells were pre-treated with IL-6 for 16 h. The blot was cut into two pieces and probed with antibody against p53 and GAPDH separately. Lower panel: quantification of p53 levels with or without doxorubicin treatment in the presence of IL-6 (S.E.M. is indicated by bars; *N* = 4). **c** mRNA levels of the p53 target gene Bax in LNCaP cells after treatment with 1 μM doxorubicin for 8 h in the presence or absence of IL-6, as determined by qRT-PCR. Cells were pre-treated with IL-6 (0.1, 1 or 10 ng/mL) for 16 h (S.E.M. is indicated by bars; *N* = 2). **d** mRNA levels of the p53 target gene MDM2 in LNCaP cells after treatment with 1 μM doxorubicin for 8 h in the presence or absence of IL-6 as determined by qRT-PCR. Cells were pre-treated with IL-6 (0.1, 1 or 10 ng/mL) for 16 h (S.E.M. is indicated by bars; *N* = 3).

To determine if IL-6 can affect the p53 response to chemotherapeutic drugs in LNCaP cells, we analyzed the impact of IL-6 on doxorubicin-mediated induction of p53. As expected, doxorubicin induced p53 protein levels in LNCaP and 22Rv1 cells, another prostate cancer line with functional p53 (Fig. 2a, b). Treatment with IL-6 led to a marked reduction of p53 protein accumulation in doxorubicin-treated cells (Fig. 2a, b). LNCaP cells treated with increasing concentrations of IL-6 showed a dose-dependent decrease in p53 protein induction upon doxorubicin treatment. At the highest concentration of IL-6 (10 ng/mL), we observed an almost 50% reduction of p53 levels (Fig. 2a). We also observed a similar IL-6-mediated reduction of p53 levels in doxorubicin-treated 22Rv1 cells (Fig. 2b). Both cell lines express IL-6 receptors (Fig. S1a). Of the three cytokines mentioned above, only IL-6 attenuated p53 induction in doxorubicin-treated LNCaP cells as shown by western blotting (Fig. S1b).

Consistent with the observed decrease in p53 protein levels, expression of the p53 target Bax, an effector of p53-dependent apoptosis, was reduced at the mRNA level in the presence of IL-6, both with and without doxorubicin treatment (Fig. 2c). In contrast, mRNA levels of MDM2, a negative regulator of p53, increased with the highest dose of IL-6 (10 ng/mL) upon doxorubicin treatment (Fig. 2d). Interestingly, the well-known p53 target p21 (CDKN1A) was upregulated both at the mRNA and protein levels in the presence of IL-6 (Fig. S2a, b), in agreement with other studies showing that IL-6 and STAT3 can enhance p21 expression independently of p53^35,36^.

### IL-6 increases p53 degradation in prostate cancer cells

To exclude that the observed p53 attenuation was a consequence of lower drug uptake or increased drug efflux, we examined intracellular drug accumulation by flow cytometry in LNCaP cells treated with doxorubicin, as described earlier^34^, with and without IL-6. This analysis showed that IL-6 only had a minor effect on the accumulation of doxorubicin in LNCaP cells (Fig. S3a).

**Fig. 3:**
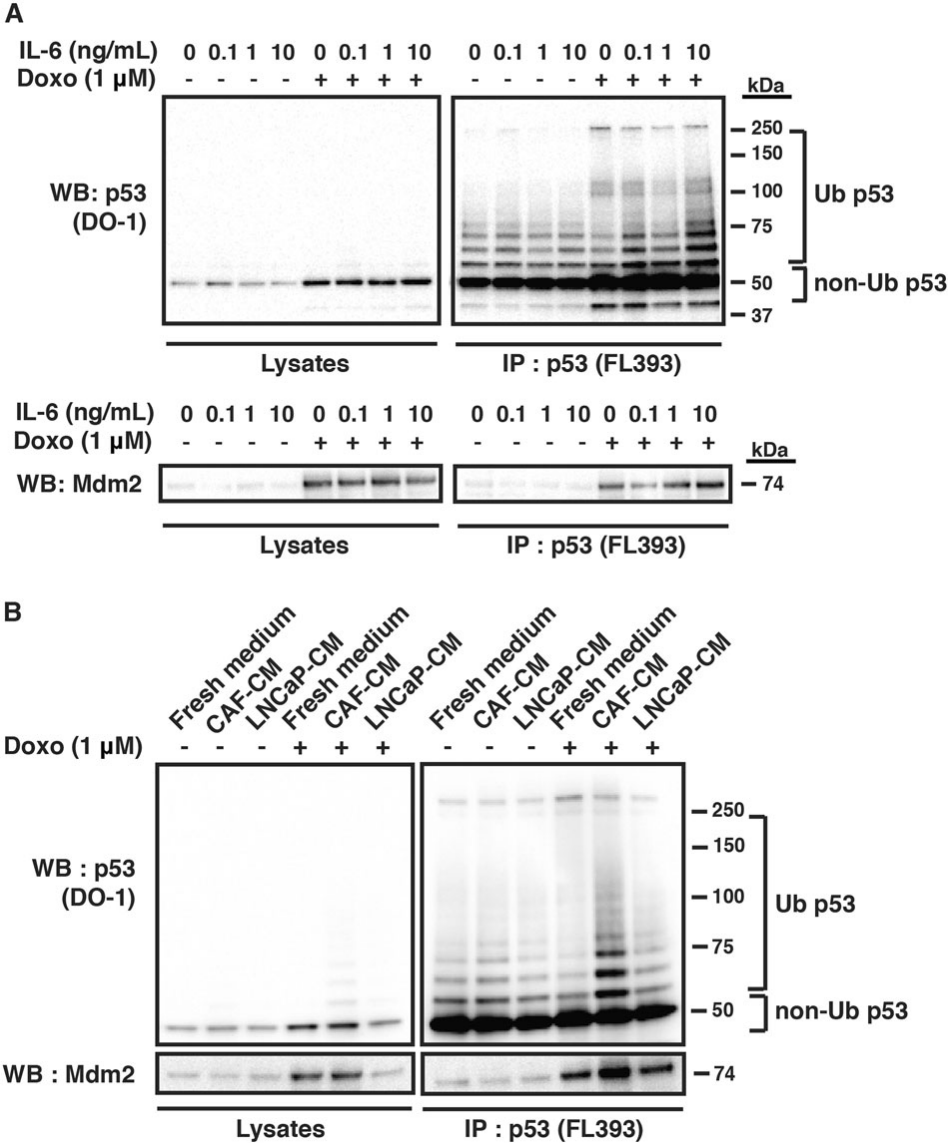
IL-6 increases p53 degradation in doxorubicin-treated prostate cancer cells. **a** Upper panel: p53 ubiquitination in lysates and p53 immunoprecipitates of LNCaP cells after treatment with 1 μM doxorubicin for 8 h in the presence or absence of IL-6 as shown by western blotting. Cells were pre-treated with IL-6 (0.1, 1 or 10 ng/mL) for 24 h. Cells were also treated with 10 μM proteasome inhibitor MG132 for 2 h prior to harvest (*N* = 1). Lower panel: Mdm2 levels in lysates and p53 immunoprecipitates of LNCaP cells after treatment with 1 μM doxorubicin for 8 h in the presence or absence of IL-6 (0.1, 1 and 10 ng/mL) as shown by western blotting. 10 μM of proteasome inhibitor MG132 was added to the cells 2 h prior to harvest (*N* = 1). **b** p53 ubiquitination in lysates and p53 immunoprecipitates of LNCaP cells after treatment with 1 μM doxorubicin for 8 h in the presence or absence of CAF-conditioned medium (CAF-CM), LNCaP-conditioned medium (LNCaP-CM), or fresh RPMI medium as shown by western blotting. Cells were pre-treated with conditioned medium for 2.5 days and with 10 μM proteasome inhibitor MG132 for 4 h prior to harvest. The blot from the same experiment also shows Mdm2 levels in lysates and p53 immunoprecipitates (*N* = 2). The blot was stripped and then blotted with an antibody against Mdm2. p53 was blotted with antibody DO-1 and immunoprecipitated with antibody FL393. Mdm2 was blotted with antibody IF2.

We next tested if IL-6 had any effect on p53 mRNA levels or p53 protein ubiquitination that regulates p53 degradation in the doxorubicin-treated LNCaP cells. Our RT-PCR analysis did not demonstrate any effect of IL-6 on p53 mRNA levels after 8 h of treatment with doxorubicin (Fig. S3b). However, we observed increased levels of ubiquitinated p53 in the presence of IL-6 after treatment of LNCaP cells with doxorubicin (Fig. 3a upper panel), as assessed by immunoprecipitation and western blotting using two different p53 antibodies. A ladder of p53 bands with slower migration is evident on the blot, indicating polyubiquitination of p53^37^. Similar results were observed in LNCaP cells cultured with CAF-conditioned medium after doxorubicin treatment (Fig. 3b). This suggests that the decreased levels of p53 protein can be attributed to increased ubiquitination and proteasomal degradation. In line with this finding, we also observed a slight increase in the levels of Mdm2 protein bound to p53 after doxorubicin treatment in the presence of IL-6 or CAF-conditioned medium (Fig. 3a lower panel, b). Thus, our results indicate that IL-6 directly stimulates p53 protein ubiquitination and degradation, leading to lower levels of p53 expression.

### IL-6 enhances prostate cancer cell survival

**Fig. 4:**
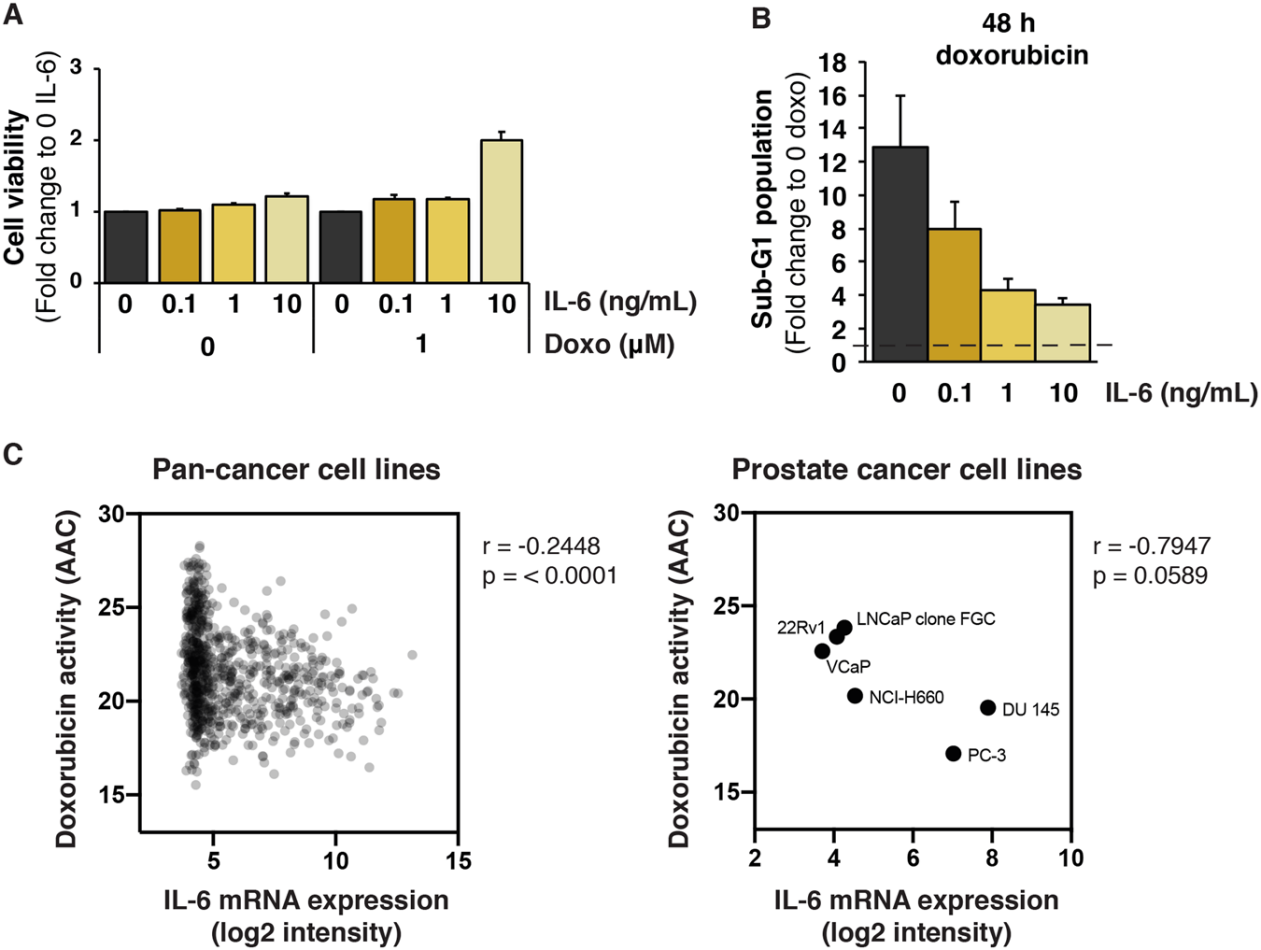
IL-6 enhances LNCaP cell survival upon doxorubicin treatment. **a** Survival of LNCaP cells after treatment with 1 µM doxorubicin for 48 h in the presence or absence of IL-6 as determined by the WST assay. Cells were pre-treated with IL-6 (0.1, 1 or 10 ng/mL) for 16 h. Cell survival is shown as fold change relative to survival in the absence of IL-6 (S.E.M. is indicated by bars; *N* = 5). **b** LNCaP cell death or sub-G1 cell population after treatment with 1 µM doxorubicin for 48 h in the presence or absence of IL-6, as assessed by PI staining and flow cytometry. Cells were pre-treated with IL-6 (0.1, 1 and 10 ng/mL) for 65 h. Sub-G1 cell populations are shown as fold change relative to sub-G1 fractions in the absence of doxorubicin (S.E.M. is indicated by bars; *N* = 5). **c** Pearson correlation (*r*) between IL-6 mRNA expression (in log2) and doxorubicin sensitivity, left panel: as shown in a panel of 796 cancer cell lines of various origin (*r* = −0.2448, *p* < 0.0001), and right panel: in 6 prostate cancer cell lines (*r* = −0.7947, *p* = 0.0589). Each dot represents doxorubicin activity for each cancer lineage. Drug sensitivity is displayed by area above the curve (AAC).

To investigate the biological effect of IL-6 upon doxorubicin treatment, we assessed LNCaP cell viability using the WST1 assay in the presence and absence of IL-6 and doxorubicin. IL-6 increased cell viability in a dose-dependent manner, both in the absence and presence of doxorubicin (Fig. 4a). This is consistent with the ability of IL-6 to stimulate tumor cell proliferation^23^. However, we observed that IL-6 at the highest concentration (10 ng/mL), increased cell viability by about two-fold in the presence of 1 µM doxorubicin (Fig. 4a), and a substantial increase was also observed at 0.5 µM doxorubicin (Fig. S4a). In the absence of doxorubicin, the effect of IL-6 was much more modest (Fig. 4a).

We also assessed the induction of cell death by flow cytometry and PI staining (Figs. 4b and S4b). We found that IL-6 caused a marked dose-dependent inhibition of the number of LNCaP cells with sub-G1 DNA content upon 48 h of treatment with doxorubicin, confirming a cytoprotective effect of IL-6. Taken together, our results show that IL-6 enhances LNCaP cell survival upon doxorubicin treatment.

To further evaluate the role of IL-6 in doxorubicin sensitivity, we analyzed IL-6 mRNA expression datasets available at the Cancer Therapeutics Response Portal (CTRP)^38^. Increased expression of IL-6 correlated with decreased sensitivity to doxorubicin treatment in cell lines representing all cancer types (Fig. 4c, left panel). A similar correlation was observed in 6 prostate cancer cell lines, although the data did not reach statistical significance (Fig. 4c, right panel). These data support the notion that IL-6 can enhance doxorubicin resistance.

### IL-6 stimulates p53 attenuation via STAT3

**Fig. 5:**
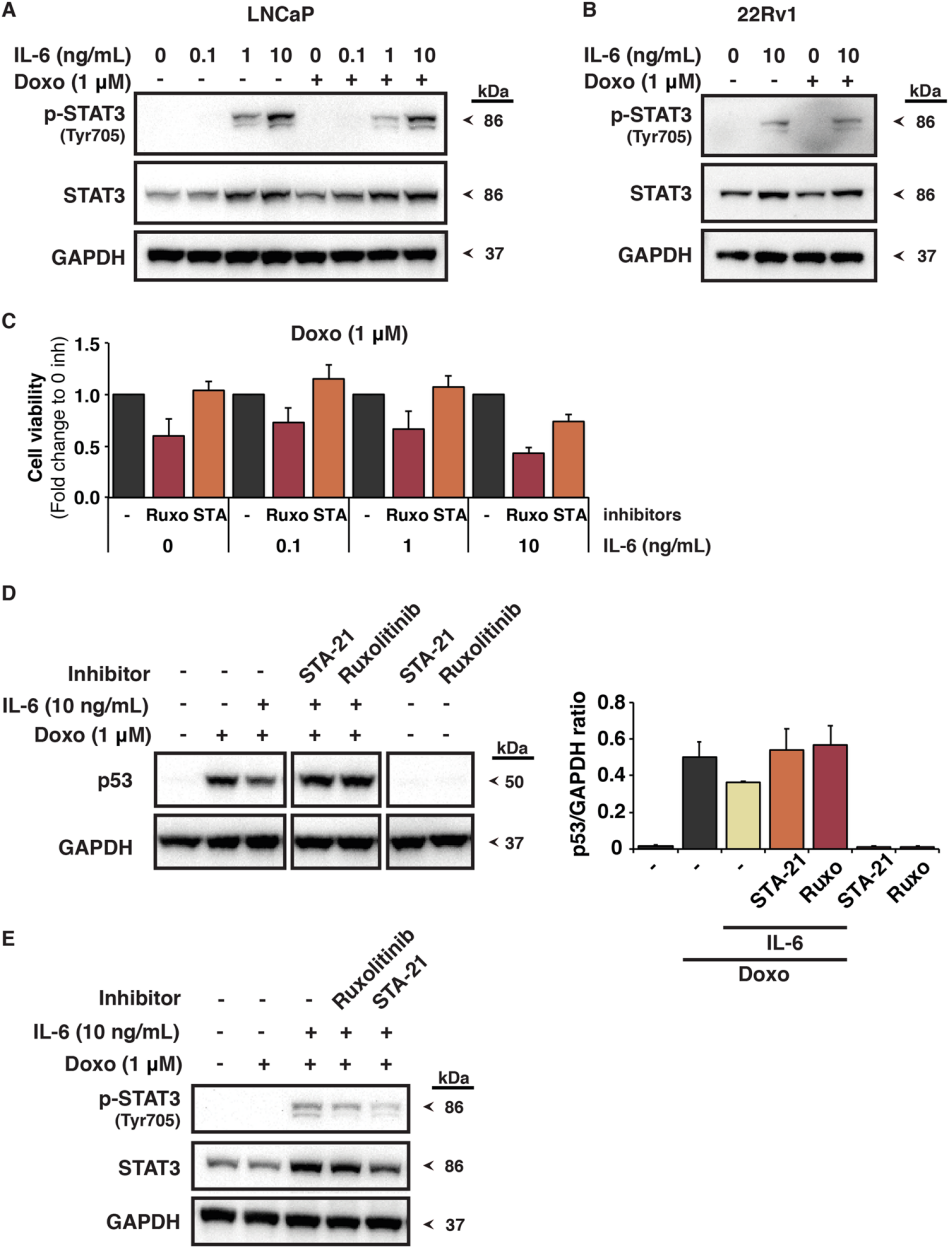
IL-6 attenuates p53 induction in response to doxorubicin via STAT3. **a** Levels of Tyr705-phosphorylated STAT3 (p-STAT3) and total STAT3 in LNCaP cells after treatment with 1 μM doxorubicin for 8 h in the presence or absence of IL-6 as shown by western blotting. Cells were pre-treated with IL-6 (0.1, 1 or 10 ng/mL) for 16 h (*N* = 5). **b** Levels of Tyr705-phosphorylated STAT3 (p-STAT3) in 22Rv1 cells after treatment with 1 μM doxorubicin for 8 h in the presence or absence of IL-6 as shown by western blotting. Cells were pre-treated with 10 ng/mL IL-6 for 16 h (*N* = 4). STAT3 phosphorylation at Tyr705 indicates STAT3 activation upon IL-6 treatment. The blots in **a**, **b** were cut into two pieces and probed with antibodies against p-STAT3 and GAPDH separately. The p-STAT3 blots were then stripped and blotted with an antibody against STAT3. **c** Survival of LNCaP cells as assessed by the WST assay after treatment with 1 μM doxorubicin for 48 h in the presence or absence of IL-6 and 1 μM JAK kinase inhibitor Ruxolitinib or 20 μM STAT3 inhibitor STA-21. Cells were pre-treated with IL-6 (0.1, 1 or 10 ng/mL) for 16 h. Cell survival is shown as fold change relative to cell survival in the absence of JAK/STAT3 inhibitor (S.E.M. is indicated by bars; *N* = 6). **d** Left panel: p53 levels in LNCaP cells after treatment with 1 μM doxorubicin for 8 h in the presence or absence of IL-6 and 1 μM JAK kinase inhibitor Ruxolitinib or 20 μM STAT3 inhibitor STA-21. Cells were pre-treated with IL-6 (10 ng/mL) for 16 h. The blot was cut into two pieces and probed with anti-p53 DO-1 and anti-GAPDH antibody separately. Samples to be compared were loaded on the same gel and transferred onto the same membrane. Right panel: quantification of the western blot data shown as ratio of p53 to GAPDH (S.E.M. is indicated by bars; *N* = 2). **e** STAT3 protein phosphorylation and total STAT3 protein levels in LNCaP cells after treatment with 1 μM doxorubicin for 8 h in the presence or absence of IL-6 and 1 μM JAK kinase inhibitor Ruxolitinib or 20 μM STAT3 inhibitor STA-21. Cells were pre-treated with 10 ng/mL IL-6 for 16 h (*N* = 2). The blot was cut into two pieces and probed with antibodies against p-STAT3 and GAPDH separately. The p-STAT3 blot was then stripped and blotted with anti-STAT3 antibody.

The JAK/STAT signaling pathway is activated in IL-6-treated cells. IL-6 induces activation of STAT3 by tyrosine phosphorylation mediated by the JAK kinase^28^. We, therefore, assessed STAT3 activation using a specific antibody against phosphorylated Tyr705, and found that STAT3 was indeed phosphorylated in both LNCaP and 22Rv1 cells treated with IL-6 (Fig. 5a, b). Likewise, STAT3 was activated in LNCaP cells cultured with CAF-conditioned medium or conditioned medium of normal fibroblasts from prostate cancer patients (NF-CM) (Fig. S5a). The activity of NF-CM in this assay is consistent with our previous results, indicating that the NFs have CAF-like features^34^ and produce IL-6 (data not shown).

Next, we investigated whether JAK/STAT3 inhibition would enhance the cytotoxic effects of doxorubicin and also restore p53 expression. We tested several inhibitors of the JAK/STAT signaling pathway and selected the JAK1/JAK2 inhibitor Ruxolitinib and the STAT3 inhibitor STA-21 that showed the most pronounced effect. In the presence of IL-6 and doxorubicin both inhibitors enhanced growth suppression (Figs. 5c and S5b), which was associated with increased accumulation of p53 protein (Figs. 5d and S5c). Neither inhibitor alone activated p53 (Fig. 5d). STAT3 phosphorylation decreased after treatment with either inhibitor in the presence of IL-6, and STA-21 also decreased the total level of STAT3 (Fig. 5e). The JAK inhibitor was more effective counteracting the protective effect of IL-6. Thus, the results from these experiments indicate that the effect of IL-6 on p53 accumulation and cell viability is at least partly mediated by JAK/STAT3 signaling.

### Database analysis of the IL-6/JAK/STAT3 pathway, MDM2, and TP53 in prostate cancer

**Fig. 6:**
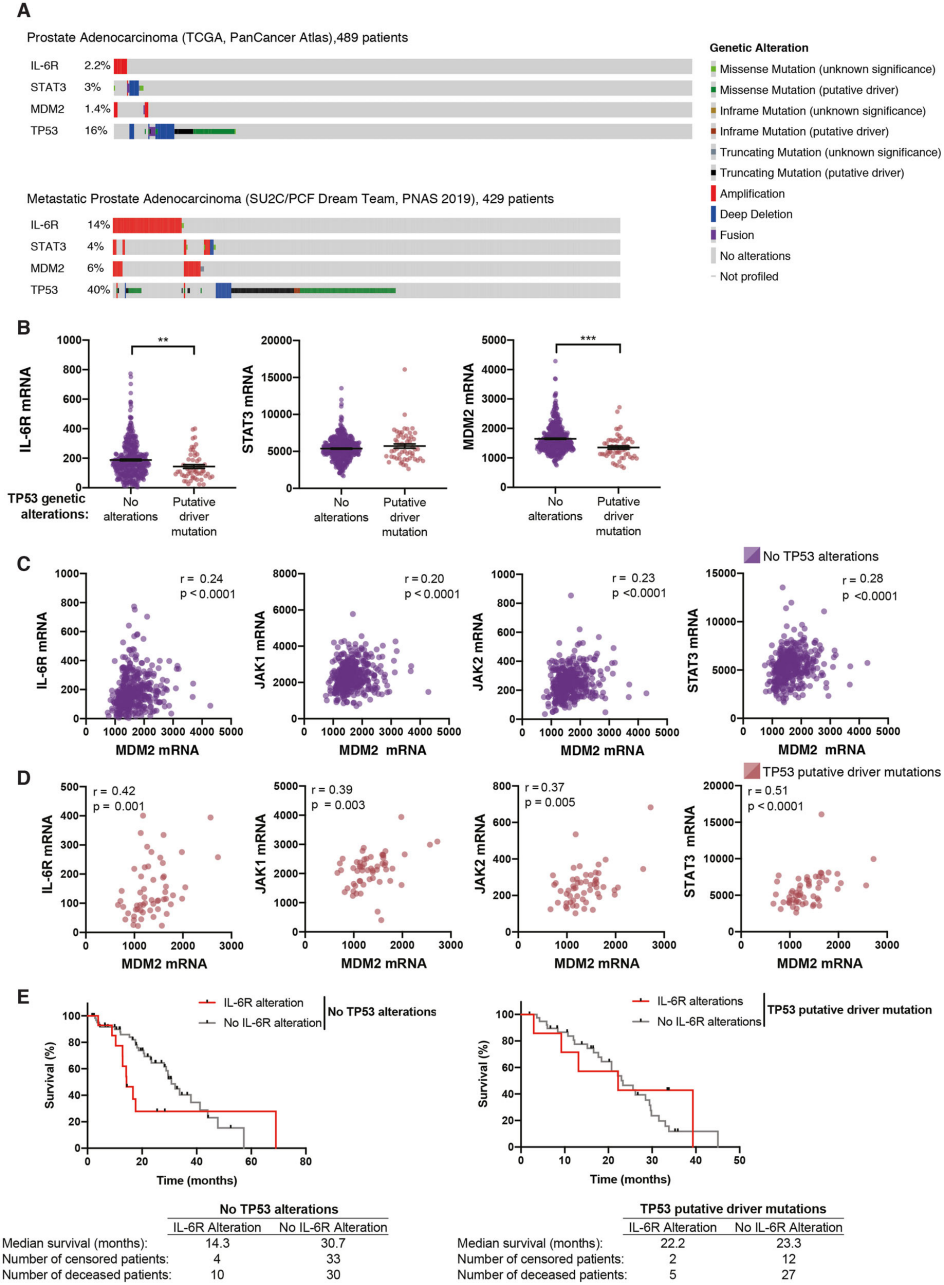
Database analysis of the IL-6/JAK/STAT3 pathway, MDM2, and TP53 in prostate cancer. **a** Oncoprint from cBioportal of two selected prostate cancer studies showing genetic alteration profiles of IL-6R, STAT3, MDM2, and TP53. Each row represents the genetic alteration according to the figure legend with individual patient in the column. **b** mRNA Expression, RSEM (Batch normalized from Illumina HiSeq_RNASeqV2) of IL-6R, STAT3 or MDM2 from prostate adenocarcinoma (prad) of the TCGA PanCancer Atlas study based on patients that have no TP53 alterations or putative driver TP53 mutations (missense or truncating). *N* = 414 no alterations, and *N* = 56 putative driver mutations. Mann–Whitney test, for IL-6R **p = 0.003, STAT3 *p* = 0.47 and MDM2 ***p < 0.0001. **c** Scatter plots of mRNA Expression, RSEM (Batch normalized from Illumina HiSeq_RNASeqV2) of IL-6R, JAK1, JAK2, STAT3 vs MDM2 in prostate adenocarcinoma patients of the TCGA PanCancer Atlas study that have no alterations in TP53. Indicated *r* and *p* values are analysis by Spearman correlation. *N* = 414 in all plots. **d** Scatter plots of mRNA expression, RSEM (Batch normalized from Illumina HiSeq_RNASeqV2) of IL-6R, JAK1, JAK2, STAT3 vs MDM2 in prostate adenocarcinoma patients of the TCGA PanCancer Atlas study that have putative driver alterations (missense and truncating) in TP53. Indicated *r* and *p* values are analysis by Spearman correlation. *N* = 56 in all plots, except the correlation with JAK2 *N* = 55. **e** Survival of patients with metastatic prostate adenocarcinoma from the study by Abida et al.^41^. *N* is indicated in the figure. Black dot indicates censored patient. Left panel; patients with no TP53 alterations were grouped as having IL-6R alterations (amplification (*N* = 12), mRNA high (*N* = 1) missense mutation (*N* = 1)) or having no IL-6R alterations. Log-rank (Mantel–Cox) test *p* = 0.09 or Gehan–Breslow–Wilcoxon test **p = 0.01. Right panel; patients with putative driver TP53 mutations (missense, truncating, inframe) were grouped as having IL-6R alterations (amplification (*N* = 4), mRNA high (*N* = 3)) or having no IL-6R alterations. Log-rank (Mantel–Cox) test *p* = 0.5 or Gehan–Breslow–Wilcoxon test *p* = 0.9.

As IL-6 induces MDM2 mRNA levels in the presence of doxorubicin (Fig. 2d) we further investigated the potential role of STAT3 in regulating MDM2 using publicly available datasets at cBioportal^39,40^. The oncoprints of prostate adenocarcinoma (from the TCGA PanCancer study) and metastatic castration-resistant prostate cancer from the study by Abida et al. show the distribution of amplification of the IL-6 receptor (IL-6R), STAT3, and MDM2 genes in prostate tumors that carry unaltered and altered TP53 (Fig. 6a)^41,42^. IL-6R amplification was more frequent in metastatic prostate adenocarcinomas and was most common in patients with unaltered TP53 (16.4%) compared to patients with altered TP53 (9.2%) (Table S1). STAT3 and MDM2 amplification was also more frequent in metastatic prostate adenocarcinomas with unaltered TP53 as compared to tumors with altered TP53 (Table S1). Analysis of The Cancer Genome Atlas (TCGA) PanCancer dataset^42^ revealed higher expression of IL-6R and MDM2 mRNA in prostate adenocarcinomas with unaltered TP53 than in tumors with putative driver TP53 mutation, while STAT3 mRNA expression levels showed no difference depending on TP53 status (Fig. 6b). These findings suggest a selection pressure for p53 inactivation via IL-6R activation and MDM2 in TP53 wild type prostate tumors.

Further analysis of the TCGA dataset revealed significant correlations between IL-6R, JAK1, JAK2, and STAT3 mRNA levels with MDM2 mRNA levels in prostate adenocarcinomas carrying unaltered TP53 (Fig. 6c). Similar correlations were also observed in prostate adenocarcinomas with putative driver mutations of TP53 (Fig. 6d). Moreover, wild type TP53 tumors with MDM2 amplification tend to express lower levels of IL-6R mRNA (Fig. S6a), suggesting that activation of the IL-6R pathway may be redundant in tumors which already have high levels of MDM2. Taken together, these results are consistent with the idea that MDM2 is upregulated by the IL-6/JAK/STAT3 pathway to attenuate the p53 response and promote chemoresistance in prostate cancers with wild type TP53.

Interestingly, our analysis of data from the study by Abida et al.^41^ indicated that patients with metastatic castration-resistant prostate cancer with unaltered TP53 and IL-6R alterations (mRNA high, amplification or missense mutation) have lower overall survival. The median overall survival for patients with IL-6R alteration was 14.3 months, compared to 30.7 months for patients without IL-6R alteration (Fig. 6e, left panel). There was no significant difference in survival depending on IL-6R status in tumors with putative TP53 driver mutations (Fig. 6e, right panel).

## Discussion

**Fig. 7:**
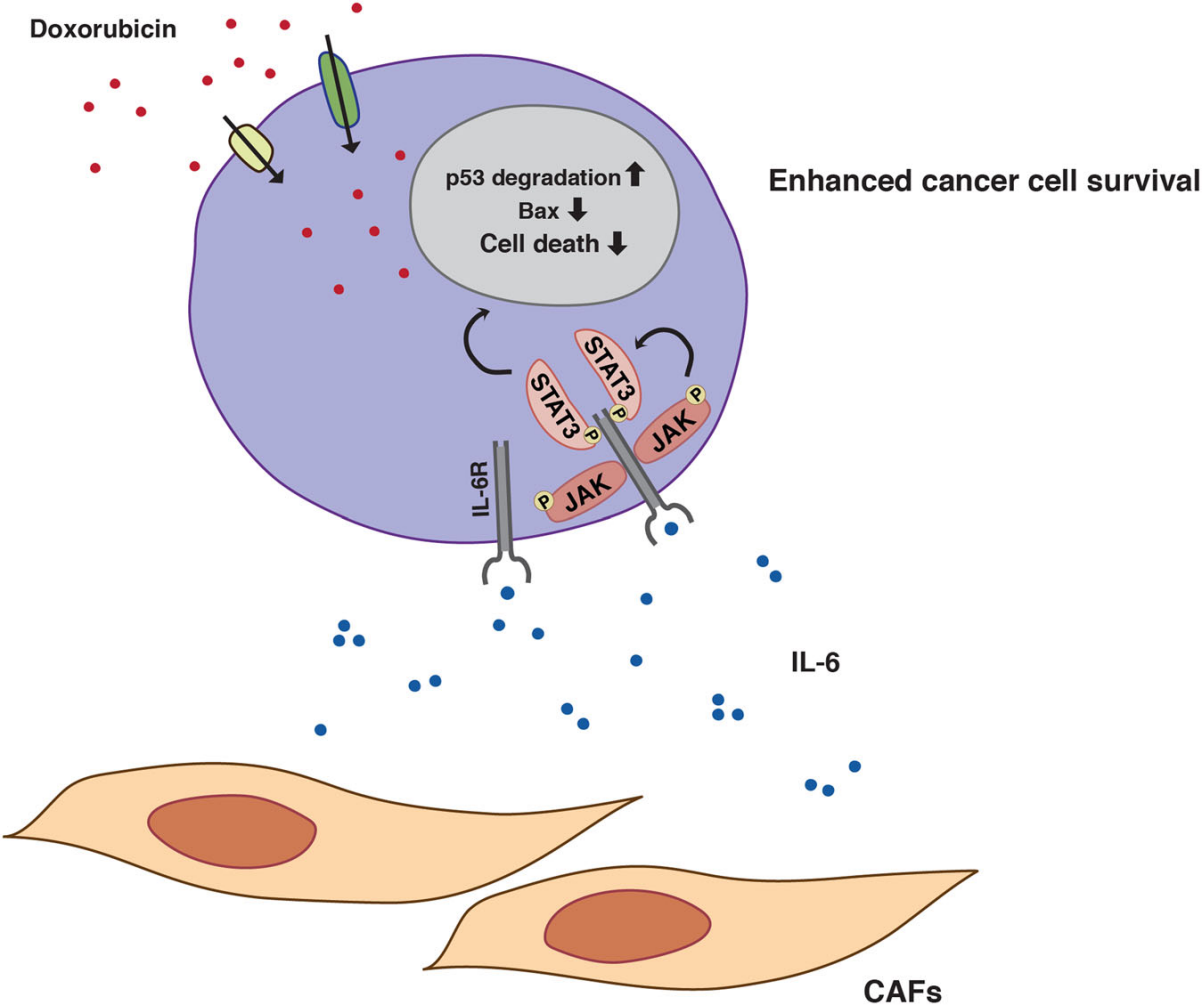
Model for CAF-mediated stimulation of prostate cancer cell chemoresistance through IL-6 and the JAK/STAT3 signaling pathway. CAFs secrete IL-6, which binds to IL-6 receptors (IL-6R) expressed on cancer cells. IL-6 binding to its receptor leads to activation of JAK kinase and STAT3, respectively. STAT3 is activated by phosphorylation at Tyr705, which induces dimerization, nuclear translocation, and DNA binding. This stimulates Mdm2-mediated p53 ubiquitination and p53 degradation in the proteasome, thereby inhibiting upregulation of the p53 target Bax upon treatment with doxorubicin. As a result, prostate cancer cell survival is enhanced.

It is well established that CAFs can support tumor growth and protect tumor cells from chemotherapy. This crosstalk is complex and only partially understood. We and others have previously found that CAFs or other stromal cells can suppress chemotherapy-induced cancer cell death by secretion of thiol-containing molecules that enhance glutathione levels in cancer cells^34,43,44^. Here we show that CAFs also produce the cytokine IL-6 and that IL-6 attenuates p53 induction and cell death upon treatment of prostate cancer cells with the chemotherapeutic agent doxorubicin. We show that IL-6 attenuates upregulation of the pro-apoptotic p53 target gene Bax, which may explain the decreased cell death observed in the presence of IL-6. Previous work has established that IL-6 can enhance tumor cell proliferation, survival, and chemoresistance^23^. Our results indicate that at least one mechanism for IL-6-mediated chemoresistance is attenuation of the p53 response. This inhibitory effect seems to be mediated through activation of STAT3 and increased Mdm2-mediated p53 ubiquitination and proteasomal degradation (Fig. 7).

Thus, accumulating evidence indicates that CAFs can protect cancer cells from cell death through multiple mechanisms. It is likely that the combined effect of thiol-containing molecules, cytokines like IL-6, and presumably other soluble factors, leads to significantly increased resistance against chemotherapeutic drugs such as doxorubicin and taxol. This is consistent with our finding that depletion of IL-6 from CAF-conditioned medium did not completely abolish the protective effect on LNCaP cells (data not shown).

A series of recent studies imply the existence of functionally distinct subsets of CAFs identified through for example single cell RNA sequencing, multi-marker FACS or lineage tracing^45–50^. The CAFs used in the present study are unselected fibroblast-like cells outgrown from primary prostate tumor tissue after radical prostatectomy^34,51^. Future studies should investigate if IL-6 mediated modulation of chemoresistance is a trait that can be assigned to emerging specific CAF subtypes such as inflammatory CAFs (iCAFs), myofibroblastic CAFs (myCAFs), CAF subsets S1-S4, antigen-presenting CAFs, vascular CAFs (vCAFs) and mCAFs from metastatic sites^45–50^.

The findings of the present study are in agreement with previous studies showing that IL-6 produced by the tumor cells or the tumor microenvironment can enhance resistance to chemotherapy in different tumor types^23^. Activation of JAK and STAT3 by IL-6 leads to increased cell survival via several pathways. Our data are also in line with a recent study showing that IL-6 produced by CAFs can enhance the chemoresistance of gastric cancer cells by activation of STAT3^52^. High IL-6 expression in resected gastric cancer tissue is correlated with poor chemotherapy response in patients. However, the study by Ham and colleagues did not address a potential role of p53 in the cytoprotective effect of IL-6. Interestingly, the observed effect was abolished by the monoclonal antibody tocilizumab against the IL-6-receptor, highlighting the IL-6/JAK/STAT3 pathway as a possible therapeutic target in cancer. A number of agents that target IL-6, the IL-6 receptor or STAT3 are now being tested in clinical trials in various types of cancer^28^.

Our analyses of publicly available datasets provided further information about the IL-6/JAK/STAT pathway and MDM2 in prostate cancers carrying wild type or mutant TP53. Amplification of the IL-6 receptor (IL-6R), STAT3 and MDM2 genes is more common in metastatic prostate adenocarcinomas that carry unaltered TP53. We also found that IL-6R and MDM2 mRNA levels are higher in wild type TP53 tumors than in tumors with putative driver TP53 mutations (Fig. 6 and Table S1). This is consistent with the idea that activation of the IL-6/JAK/STAT3 pathway and MDM2 represents an alternative mechanism for p53 inactivation during tumor progression. However, STAT3 mRNA expression levels did not correlate with TP53 status. One possible explanation is the frequent activation of STAT3 in cancer by STAT3 protein phosphorylation^28,53^. Thus, STAT3 activity may not always correlate directly with STAT3 mRNA levels. The observed correlation between IL-6R/JAK/STAT3 and MDM2 mRNA levels in prostate tumors regardless of TP53 status could reflect the fact that Mdm2 also has p53-independent oncogenic effects and is overexpressed in mutant TP53 tumors as well^54^.

Mdm2 is known to be regulated by phosphorylation by kinases such as ATM and Akt^55,56^. ATM-mediated Mdm2 phosphorylation leads to p53 activation, while Akt enhances Mdm2-mediated p53 ubiquitination and degradation via phosphorylation of Mdm2 Ser166. Akt is downstream of JAK in the IL-6/PI3K/Akt pathway, and this pathway has been shown to promote prostate cancer cell survival^57,58^. However, we did not observe any effect of IL-6 on Mdm2 Ser166 phosphorylation in our LNCaP cells (data not shown), suggesting that Ser166 phosphorylation is not required for IL-6-induced attenuation of p53. Mdm2 is not known as a direct target of STAT3 and so our findings raise the question as to how IL-6/JAK/STAT3 might stimulate Mdm2-mediated p53 ubiquitination. Interestingly, MDM2 has been shown to be upregulated at the mRNA and protein levels by Leukemia inhibitory factor (LIF), a member of the IL-6 family of cytokines, and this effect appears to be mediated by STAT3^59^. This supports our idea that CAF-derived IL-6 can upregulate MDM2 via STAT3, leading to an attenuated p53 response to doxorubicin.

In conclusion, secretion of the cytokine IL-6 is one important mechanism by which CAFs can enhance chemoresistance of tumor cells. IL-6 affects p53 turnover and expression levels via JAK/STAT3. Further studies of how CAFs affect the p53 response to DNA-damaging chemotherapeutic drugs in tumor cells may provide novel opportunities for efficient cancer therapy aimed at blocking pro-survival signaling from the tumor microenvironment.

## Materials and methods

### Cell culture

LNCaP and 22Rv1 cells were purchased from the American Type Culture Collection (ATCC, Manassas, VA, USA) and grown in RPMI 1640 medium containing 2.05 mM L-glutamine (HyClone, Logan, UT, USA) supplemented with 10% fetal bovine serum (HyClone) and 2.5 μg/mL plasmocin (InvivoGen, San Diego, CA, USA). LNCaP cells carry wild type TP53 (p53^wt/wt^)^60^. 22Rv1 cells carry one wild type TP53 allele and one allele with the Q331R mutation (p53^wt/mut^). 22Rv1 cells retain partial p53 function^61^. Primary human prostate fibroblasts were established and cultured as described^34,51^. All cells were maintained in 5% CO_2_ and 100% humidity at 37 °C, and were tested regularly and negative for mycoplasma. Conditioned media were generated as described^34^.

### Antibodies and reagents

Cells were treated with recombinant human HGF (HumanZyme, Chicago, IL, USA), OPG (Nordic BioSite, Täby, Sweden) or IL-6 (HumanZyme) and then exposed to doxorubicin (Sigma-Aldrich, Schnelldorf, Germany). The primary antibodies used in this study were anti-p53 DO-1 mouse monoclonal (Santa Cruz Biotechnology, Santa Cruz, CA, USA, Cat.# sc-126), anti-p53 FL393 rabbit polyclonal (Santa Cruz, Cat.# sc-6243), anti-p21 Waf1/Cip1 DCS60 mouse monoclonal (Cell Signaling, Leiden, The Netherlands, Cat.# 2946), anti-MDM2 IF2 mouse monoclonal (Calbiochem, Solna, Sweden, Cat.# OP46), anti-phospho-STAT3 (Tyr705) 3E2 mouse monoclonal (Cell Signaling, Cat.# 9138), anti-STAT3α D1A5 XP rabbit monoclonal (Cell Signaling, Cat.# 8768), anti-GAPDH FL-335 rabbit monoclonal (Santa Cruz, Cat.# sc-25778) and anti-IL-6Rα C-20 rabbit polyclonal (Santa Cruz, Cat.# sc-661). Secondary antibodies used were Rabbit anti-Mouse IgG Secondary Antibody, HRP (Invitrogen, Cat.# 61-6520) or Goat anti-Rabbit IgG Secondary Antibody, HRP (Invitrogen, Cat.# 65-6120). The inhibitors used were JAK inhibitors Ruxolitinib (INCB018424 Selleckchem, Rungsted, Denmark) and Pyridone 6 (Calbiochem) and STAT3 inhibitors STA-21 and Stattic (both from Selleckchem, Denmark).

### Cytokine evaluation

Conditioned media from CAFs or LNCaP cells were collected as described^34^ after 3 days of cultivation with RPMI 1640 medium and at similar cell density. The collected conditioned media were filtered through 0.2 μm membrane before the assay. Human cytokine antibody array (G-series 1000) was purchased from RayBiotech (Norcross, GA, USA) and every step was performed according to the company’s protocol. 100 μL of each conditioned medium was added to each well of the array slides. After 2 h of incubation, biotin-conjugated antibody was added to the prepared samples followed by fluorescent dye-conjugated streptavidin. The cytokines present were detected using a fluorescence detector system (ImageQuant LAS 4000 series with Cy3 detection filter (GE Healthcare, Danderyd, Sweden)).

### Enzyme-linked immunosorbent assay (ELISA)

IL-6 contents were measured by ELISA assay kit (R&D Systems, Abingdon, UK) according to the manufacturer’s instructions with each sample in triplicate. Briefly, the ELISA microplate pre-coated with antibody was incubated with conditioned media from CAFs and LNCaP cells for 2 h. Horseradish peroxidase-labeled secondary antibody was added to each well. After removal of the reaction solution, substrate solution was added to the plate. The reaction was terminated by adding stop solution and absorbance values were measured at 450 nm on a microplate reader (Molecular Device VERSAmax Tunable Microplate Reader; San Jose, CA, USA) and were subtracted from the reading at 540 nm. IL-6 contents in the supernatant were calculated in accordance with a standard curve. Average values were calculated from two or three experiments.

### Western blotting

LNCaP cells were seeded at 4 × 10^5^ cells/well and 22Rv1 at 1.5 × 10^5^ cells/well in 6-well plates with 2 mL complete medium, adhered overnight and treated with recombinant IL-6 for 16 h, unless otherwise stated, prior to doxorubicin treatment. Thereafter, medium was removed, doxorubicin was added together with fresh medium and IL-6, and cells were incubated for additional 8 h. For JAK/STAT3 inhibition, inhibitors were added at the same time as doxorubicin treatment, 8 h prior to harvesting. Whole-cell lysates were then prepared with lysis buffer containing 100 mM Tris pH 7.4, 150 mM NaCl, 1% NP 40, 1% Protease Inhibitor Cocktail (Sigma-Aldrich) and 10% Phosphatase Inhibitor Cocktail (Roche, Penzberg, Germany). The protein concentrations were determined by Bradford assay. 25 μg of proteins were heated at 95 °C for 10 min and separated by SDS-PAGE using 10% Bis-Tris polyacrylamide gels (Life Technologies, Stockholm, Sweden) in 1× MOPS buffer (Life Technologies). Proteins were then transferred to nitrocellulose membranes using iBlot2 (Life Technologies). After blocking, the membranes were incubated with primary antibodies (1:500–1:1000) at 4 °C overnight and probed with anti-mouse or anti-rabbit HRP-conjugated secondary antibody (1:10,000) the next day for 1 h at room temperature. Finally, the blots were detected with Super Signal West Femto Maximum Sensitivity Substrate (Thermo Scientific, Stockholm, Sweden) and a CCD camera (Fujifilm, Stockholm, Sweden). GAPDH served as loading control. Samples to be compared were loaded on the same gel and transferred onto the same membrane.

### Quantitative real time-PCR and Taqman gene expression assay

LNCaP cells were seeded at 4 × 10^5^ cells/well in 6-well plates with 2 mL complete medium, adhered overnight and pre-treated with recombinant IL-6 for 16 h. Thereafter, medium was removed, doxorubicin was added together with fresh medium and IL-6, and cells were incubated for additional 8 h. Cellular RNA was isolated using RNeasy mini kit (Qiagen, Hilden, Germany) according to the manufacturer’s instructions. Total RNA was then reverse-transcribed to cDNA using SuperScript II Reverse Transcriptase (Invitrogen, Stockholm, Sweden). 50 ng of the cDNA was further amplified through a quantitative real-time PCR, which was carried out in an Applied Biosystems StepOnePlus Real-Time PCR machine, using TaqMan PCR mix contained FastStart Universal Probe Master (Roche) and 1× Taqman probe in a total volume of 20 μL. TaqMan probes that were used were specific for MDM2 (Hs01066930_m1), Bax (Hs00414514_m1), p21 (CDKN1A) (Hs00355782_m1), TP53 (Hs00153340_m1) and GAPDH (Hs99999905_m1). GAPDH served as a reference gene. We also used 18S as another reference gene but detected no major difference in expressions compared to GAPDH (data not shown). Thermal cycling was conducted as follows: 50 °C 2 min, 95 °C 10 min and 40 cycles comprising 95 °C 15 s and 60 °C 1 min with fluorescence reading using FAM channel. Amplifications of the PCR products were quantified by the number of threshold cycle (C_T_), and the relative gene expression was determined by using the 2^−ΔΔCT^ comparative method. The C_T_ values for target genes were normalized to the value of GAPDH by calculating the change in C_T_ (ΔC_T_). ΔΔC_T_ values were calculated by subtracting the ΔC_T_ value for each sample from the ΔC_T_ value of the control sample.

### Immunoprecipitation

LNCaP cells were seeded in 100 × 20 mm dishes with 3 × 10^6^ cells per dish, adhered overnight, pre-treated with recombinant IL-6 for 24 h or with conditioned media for 2.5 days. The medium was then removed, doxorubicin was added together with fresh medium and IL-6 or with conditioned media, and cells were incubated for additional 8 h. 2 or 4 h prior to harvesting, cells were treated with 10 μM of the proteasome inhibitor MG132 (Sigma-Aldrich). Thereafter, cells were lysed in lysis buffer containing 100 mM Tris pH 7.4, 150 mM NaCl, 1% NP 40, 1% Protease Inhibitor Cocktail (Sigma-Aldrich) and 2 mM deubiquitinating enzyme inhibitor, N-Ethylmaleimide (NEM) (Sigma-Aldrich). Protein concentrations were determined by Bradford assay. Each supernatant containing 1 mg/mL of proteins was incubated with 1 μg anti-p53 rabbit antibody per 1 mg protein under rotation at 4 °C. After 1 h of incubation, 10 μL of Dynabeads Protein G (Life Technologies) were added into each sample and incubated overnight for immunoprecipitation of p53 proteins. After all the washing steps, proteins were separated and run for Western blotting. Antibody used for Western blotting was anti-p53 or anti-Mdm2 mouse antibody.

### WST-1 cell viability assay

Anti-proliferative effect of doxorubicin in the presence of recombinant IL-6 on cancer cells was determined using WST-1 colorimetric assay (Roche). 1.2 × 10^5^ LNCaP cells per 96-well were seeded in 100 μL medium. The next day, cells were pre-treated with IL-6 for 16 h and thereafter treated with doxorubicin for 48 h. For JAK/STAT3 inhibition, inhibitors were added at the same time as doxorubicin. After the treatments, 10 μL of WST-1 reagent was added to each well, with each sample in duplicate, and incubated at 37 °C for 30 (Fig. 5c) or 60 min (Fig. 4a). The absorbance was read using a microplate reader (TECAN infinite M1000, Tecan trading AG) at 450 nm. Absorbance values representing metabolic activity were expressed as percentage of viable cells compared to untreated cells, or recalculated and presented as fold change relative to cell survival in the absence of IL-6 or inhibitors.

### Sub-G1 assay by PI staining

2 ×10^5^ LNCaP cells were seeded in six-well plates with 2 mL complete medium. After adhesion overnight, cells were pre-treated with IL-6 for 65 h and thereafter treated with doxorubicin for 48 h. All cells were then harvested, washed with cold PBS and pelleted. The cell pellets were re-suspended in 3 mL cold PBS, and 4 mL of 99% cold ethanol was added dropwise while vortexing. The samples were stored overnight for fixation at 4 °C. Thereafter, cells were centrifuged, re-suspended, and stained with 0.05 mg/mL Propidium iodide (Sigma-Aldrich) together with 0.25 mg/mL RNAse A (Sigma-Aldrich) at 37 °C for 45 min. Additional PBS was added to each sample afterward and the cells were analyzed using BD FACSCalibur flow cytometer shortly after. Approximately 10,000 cells were assessed for each experiment.

### Immunofluorescence staining

LNCaP cells were seeded at 4 × 10^5^ cells and 22Rv1 at 2 × 10^5^ cells per well in 6-well plates with 22 × 22 mm glass slides at the bottom of each well. Cells were allowed to adhere on the glass slides overnight before the immunofluorescence staining was performed. After washing with PBS, cells were fixed with 4% formaldehyde for 10 min, permeabilized with 0.2% Triton for 2 min, and then blocked with 2% BSA in PBS-T for 15 min. The fixed cells were then incubated with the anti-IL-6 receptor primary antibody (1:100) at 4 °C overnight. The next day, cells were washed with PBS and incubated with anti-rabbit Alexa Fluor 594 conjugated secondary antibody (1:1000) (Life Technologies) for an hour at room temperature. Finally, the cells were washed with PBS and mounted with cover slips using Vectashield Hard Set Mounting Medium containing DAPI (Vector Laboratories, Burlingame, CA, USA). Images were captured on a Zeiss AxioPlan 2 microscope connected to an AxioCam HRm microscope camera. Jurkat cells that are known not to express IL-6Rα^62^ were used as negative control for IL-6 receptor.

### Cellular drug content assay

The assay was performed as described^34^. Briefly, LNCaP cells were seeded and pre-treated with different concentrations of recombinant IL-6 for 16 h. The medium was then removed, doxorubicin was added together with fresh medium and IL-6, and cells were incubated for additional 8 h. Cells were harvested after treatments and the intracellular doxorubicin accumulation was assessed using a NovoCyte flow cytometer. The cells were analyzed with excitation at 488 nm and emission integrated at 530 nm. A total 10^4^ cells were included in the analysis for each experiment.

### Database analysis and statistics

All experiments were performed independently. N is the number of times each experiment was repeated or number of cell lines or patients included in each group analyzed. The data are shown as mean and standard error of the mean (S.E.M.). For the analysis of doxorubicin sensitivity in cell lines, gene expression data from the Cancer Cell Line Encyclopedia data portal (CCLE, https://portals.broadinstitute.org/ccle)^63^ and drug sensitivity data from Cancer Therapeutics Response Portal (CTRP, https://portals.broadinstitute.org/ctrp/)^38^ were downloaded. The correlation between IL-6 mRNA expression and drug sensitivity data was then assessed using CellMinerCDB^64^. Larger area above the curve (AAC) indicates that the drug is more efficient at killing cells. Oncoprints and all patient data were downloaded from cBioportal (https://www.cbioportal.org/)^39,40^. Patients from the TCGA PanCancer Atlas Studies^42^ were selected for having genetic alterations with putative driver TP53 mutation (missense mutations or truncating mutations) or no TP53 alteration, and MDM2 amplification or no MDM2 alteration. mRNA expression, RSEM (batch normalized from Illumina HiSeq_RNASeqV2) of IL-6R, MDM2, STAT3, JAK1, JAK2 of selected patients were plotted for correlation or grouped analysis. Frequency of genetic alterations was based on patients that had been profiled for both copy number alterations and mutations in IL-6R, MDM2, STAT3 and TP53. For the survival analysis the Abida et al. study data^41^ with patients containing survival data was used. Patients without TP53 alterations or with putative TP53 driver mutations (missense, truncating or inframe) were selected for presence of IL-6R alterations (mRNA high, amplification or missense mutations) or not. All statistical tests and database analyses were generated in GraphPad Prism 8. Statistical test for survival analysis was Log-rank (Mantel–Cox) test and Gehan–Breslow–Wilcoxon test. For grouped and correlation analysis, normal and lognormal distribution was tested by Shapiro–Wilk test and D’Agostino & Pearson test. Based on outcome of normality test, Mann–Whitney test was used for grouped analysis, and for correlations, Pearson or Spearman correlation score (*r*) and *p* value were calculated.

## Supplementary information

Supplementary Figure 1

Supplementary Figure 2

Supplementary Figure 3

Supplementary Figure 4

Supplementary Figure 5

Supplementary Figure 6

Supplementary figure legends

Supplementary Table 1
