# Supplementary figures and images for: Interleukin-6 derived from cancer-associated fibroblasts attenuates the p53 response to doxorubicin in prostate cancer cells
Source: Cell Death Discov. 2020 Jun 2;6:42. doi: 10.1038/s41420-020-0272-5 (PMC7265343; doi:10.1038/s41420-020-0272-5)

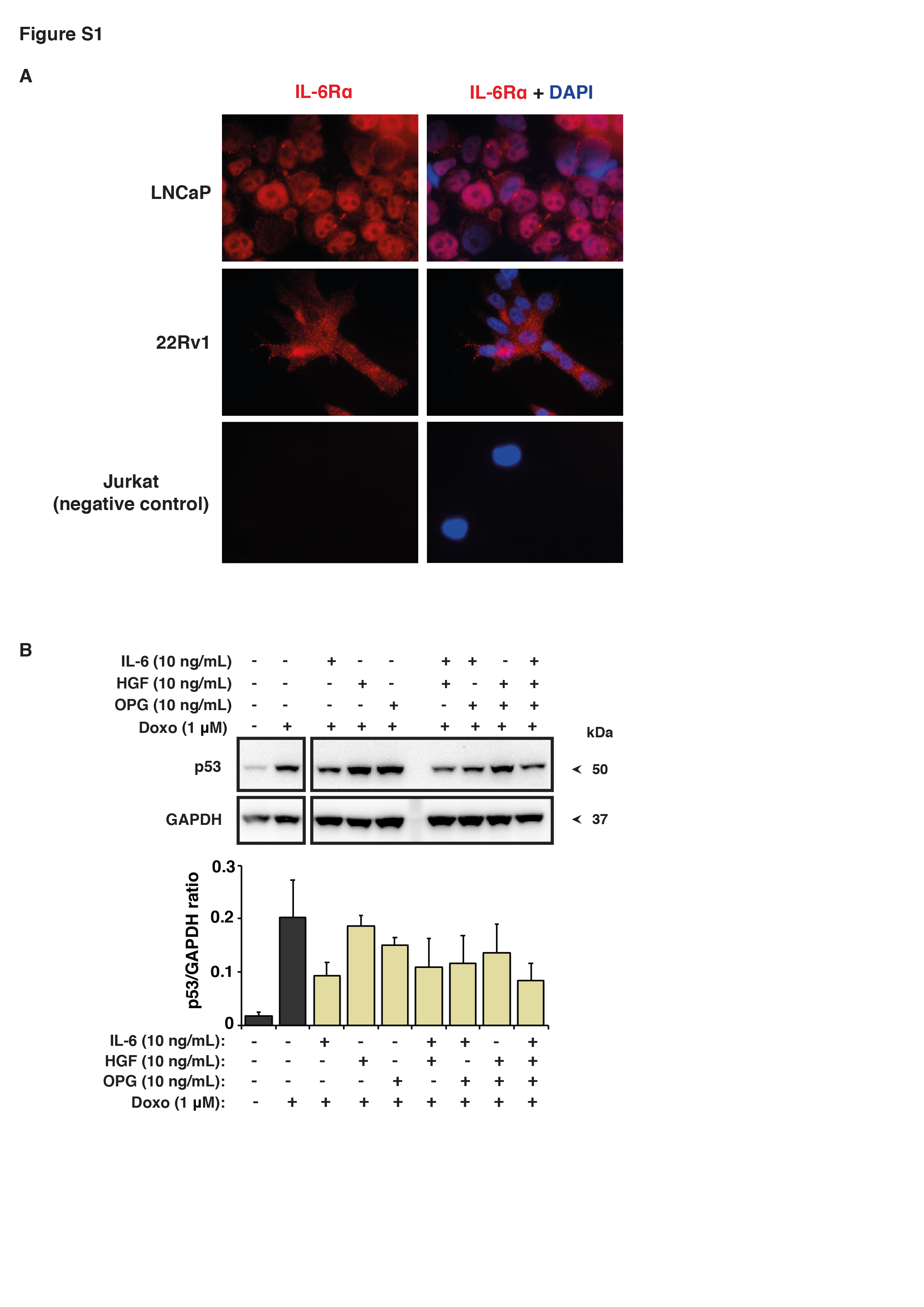

Supplement: Supplementary file 1 — Supplementary Figure 1 [file 41420_2020_272_MOESM1_ESM.png]

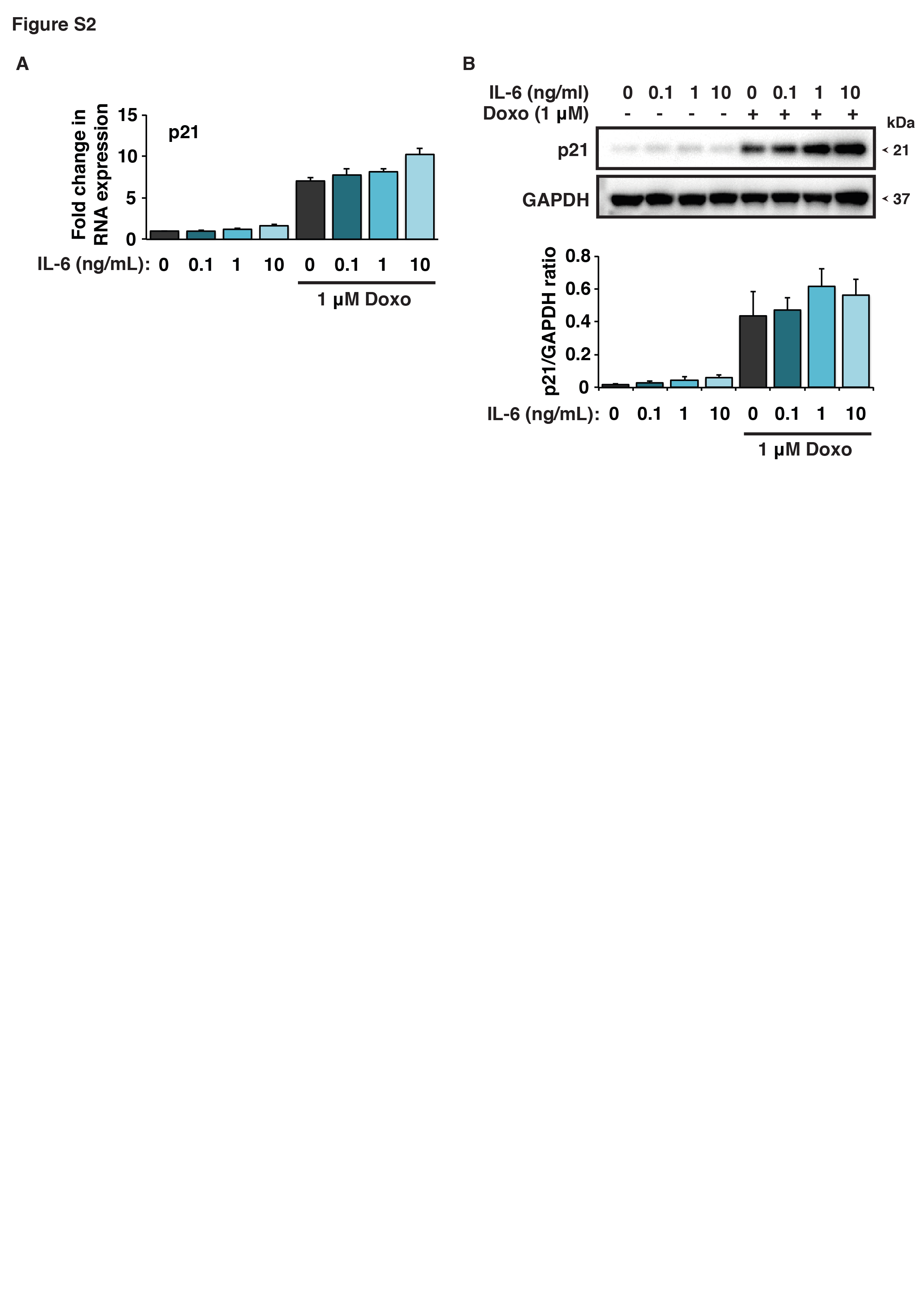

Supplement: Supplementary file 2 — Supplementary Figure 2 [file 41420_2020_272_MOESM2_ESM.png]

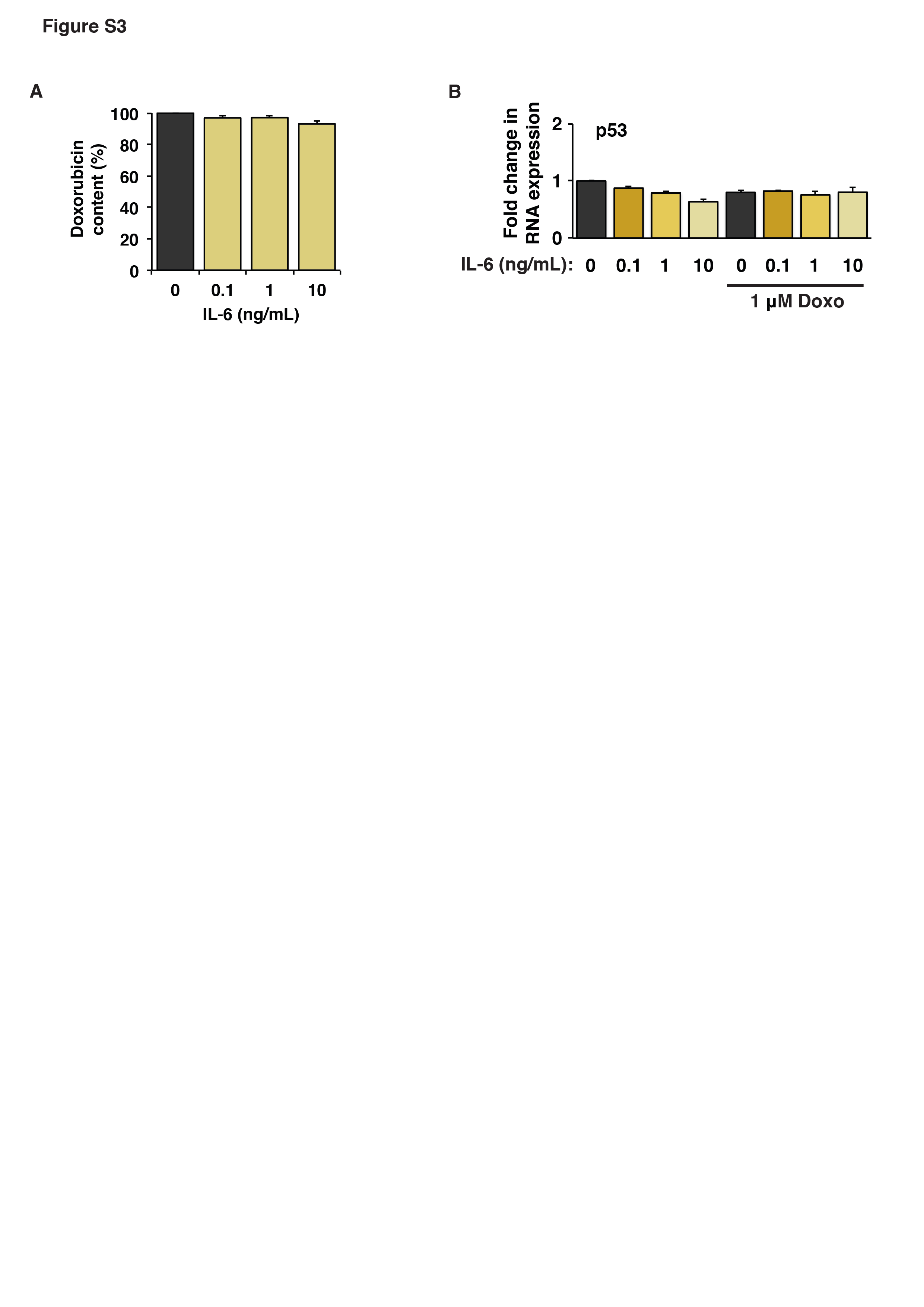

Supplement: Supplementary file 3 — Supplementary Figure 3 [file 41420_2020_272_MOESM3_ESM.png]

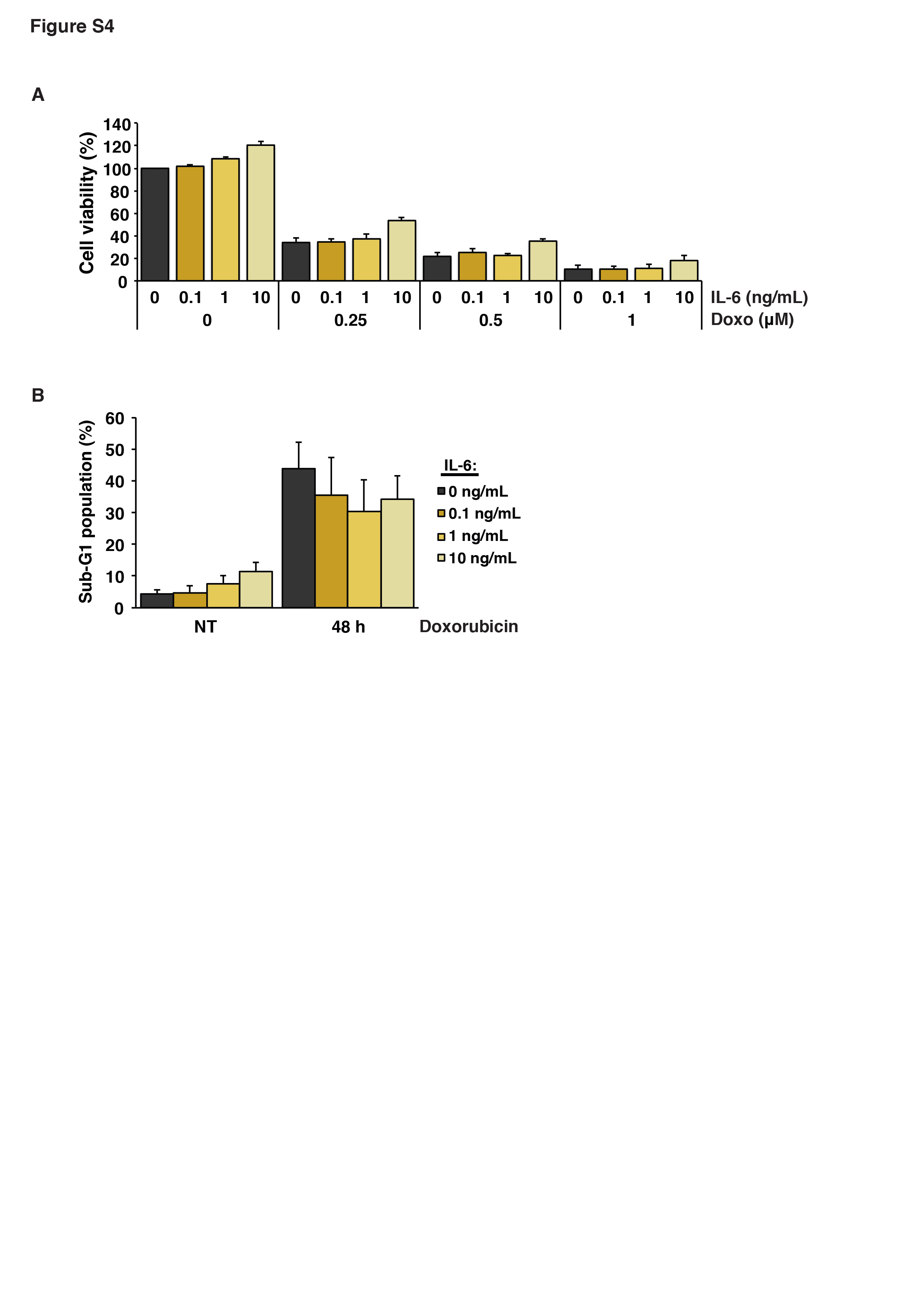

Supplement: Supplementary file 4 — Supplementary Figure 4 [file 41420_2020_272_MOESM4_ESM.png]

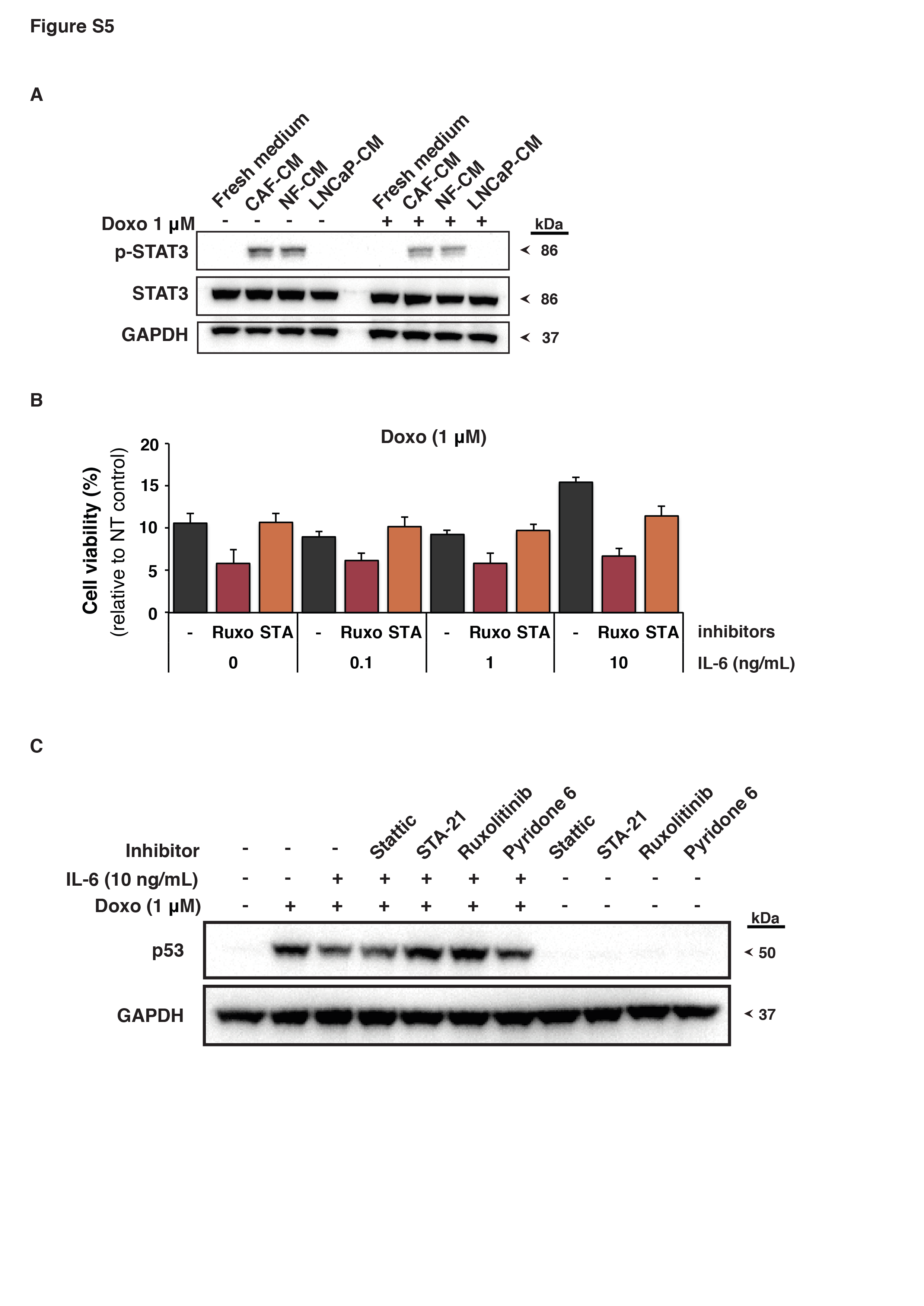

Supplement: Supplementary file 5 — Supplementary Figure 5 [file 41420_2020_272_MOESM5_ESM.png]

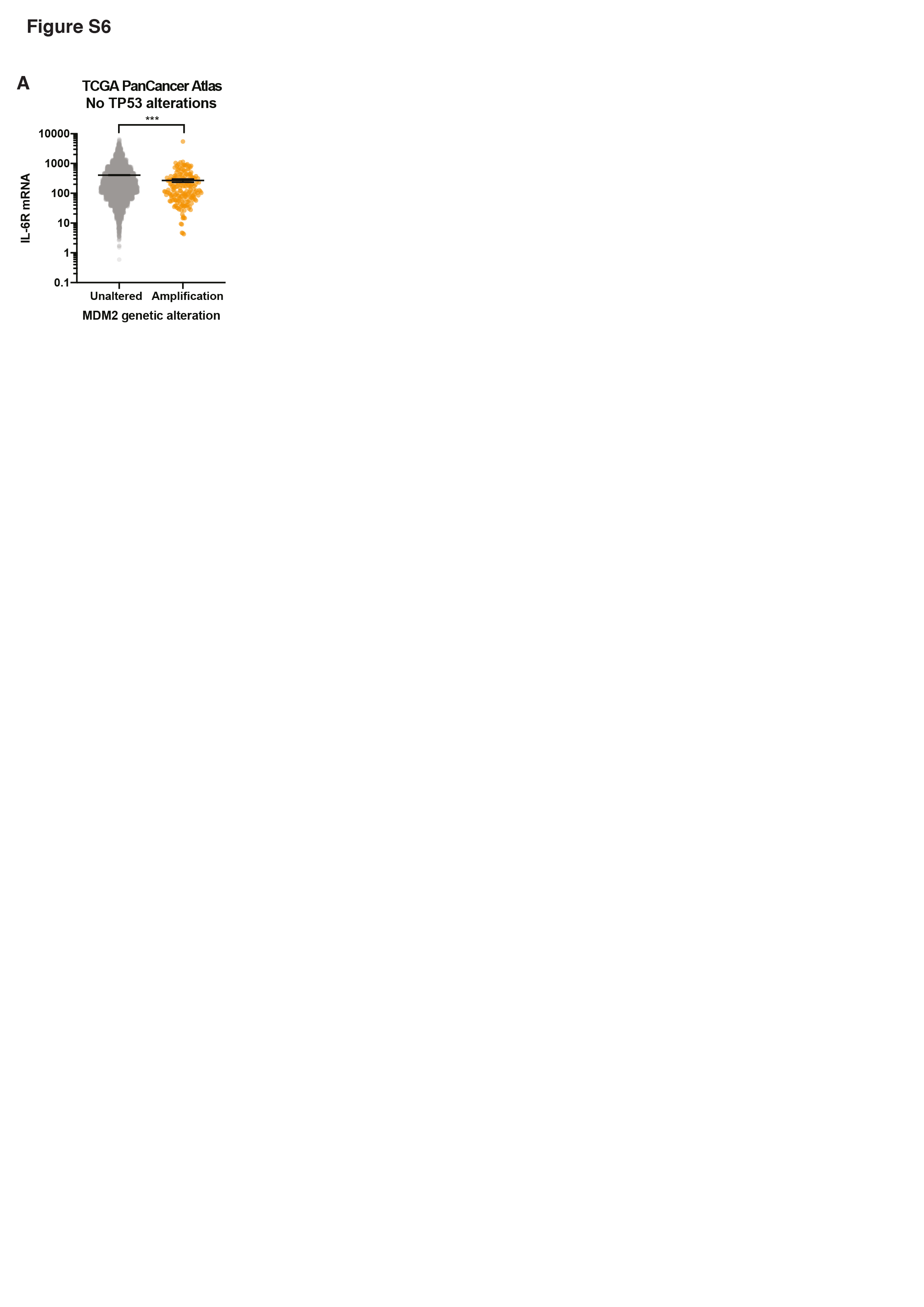

Supplement: Supplementary file 6 — Supplementary Figure 6 [file 41420_2020_272_MOESM6_ESM.png]
