# Supplementary material for: Interleukin-6 derived from cancer-associated fibroblasts attenuates the p53 response to doxorubicin in prostate cancer cells
Source: Cell Death Discov. 2020 Jun 2;6:42. doi: 10.1038/s41420-020-0272-5 (PMC7265343; doi:10.1038/s41420-020-0272-5)
Supplement: Supplementary file 7 — Supplementary figure legends [file 41420_2020_272_MOESM7_ESM.docx]

**Supplementary Information**

**Supplementary Figure Legends**

**Figure S1. Expressions of IL-6 receptor and induction of p53 in prostate cancer cells.** (**A**) Immunofluorescence staining of IL-6 receptor (red), IL-6Rα chain, in LNCaP and 22Rv1 cells (N=2). Cell nuclei were stained with DAPI (blue). Original magnification x 40. Jurkat cells served as a negative control. (**B**) Upper panel: p53 levels in LNCaP cells after treatment with 1 μM doxorubicin for 8 hours in the presence or absence of 10 ng/mL IL-6, 10 ng/mL HGF and 10 ng/mL OPG, as shown by Western blotting. Cells were pre-treated with IL-6, HGF or OPG for 65 hours. The blot was cut into two pieces and probed with anti-p53 DO-1 and anti-GAPDH antibody separately. Samples to be compared were loaded on the same gel and transferred onto the same membrane. Lower panel: quantification of the Western blot data shown as ratio of p53 to GAPDH (S.E.M. is indicated by bars; N=3).

**Figure S2. IL-6 affects the expression of p53 targets in prostate cancer cells.** (**A**) mRNA levels of the p53 target gene p21 in LNCaP cells after treatment with 1 μM doxorubicin for 8 hours in the presence or absence of IL-6, as determined by qRT-PCR. Cells were pre-treated with IL-6 (0.1, 1 or 10 ng/mL) for 16 hours (S.E.M. is indicated by bars; N=3). (**B**) Upper panel: p21 protein levels in LNCaP cells treated with same conditions, as shown by Western blotting. Lower panel: quantification of the Western blot data shown as ratio of p21 to GAPDH (S.E.M. is indicated by bars; N=3).

**Figure S3. IL-6 does not decrease p53 mRNA levels in doxorubicin-treated LNCaP cells.** (**A**) Doxorubicin content in LNCaP cells exposed to 8 hours of 1 μM doxorubicin in the presence or absence of IL-6, as assessed by flow cytometry. Cells were pre-treated with IL-6 (0.1, 1 or 10 ng/mL) for 16 hours (S.E.M. is indicated by bars; N=3). (**B**) p53 mRNA levels in LNCaP cells after treatment with 1 μM doxorubicin for 8 hours in the presence or absence of IL-6 as determined by qRT-PCR. Cells were pre-treated with IL-6 (0.1, 1 or 10 ng/mL) for 16 hours (S.E.M. is indicated by bars; N=4).

**Figure S4. IL-6 enhances LNCaP cell survival upon doxorubicin treatment.** (**A**) Survival of LNCaP cells after treatment with doxorubicin (0.25, 0.50 and 1.0 µM) for 48 hours in the presence or absence of IL-6 as determined by the WST assay. Cells were pre-treated with IL-6 (0.1, 1 or 10 ng/mL) for 16 hours. Cell survival is shown as percentage of viable cells compared to untreated cells (S.E.M. is indicated by bars; N=5). (**B**) LNCaP cell death after treatment with 1 µM doxorubicin for 48 hours in the presence or absence of IL-6, as assessed by PI staining and flow cytometry. Cells were pre-treated with IL-6 (0.1, 1 and 10 ng/mL) for 65 hours. Cell death is shown as percentage of sub-G1 cell populations (S.E.M. is indicated by bars; N=5).

**Figure S5. IL-6 attenuates p53 induction in response to doxorubicin via STAT3. (A)** Levels of Tyr705-phosphorylated STAT3 (p-STAT3) and total STAT3 in LNCaP cells after treatment with 1 μM doxorubicin for 8 hours in the presence or absence of CAF-conditioned medium (CAF-CM), conditioned medium of normal fibroblasts from prostate cancer patients (NF-CM), LNCaP-conditioned medium (LNCaP-CM), or fresh RPMI medium as shown by Western blotting. Cells were pre-treated with conditioned media for 2.5 days (N=3). (**B**) Survival of LNCaP cells as assessed by the WST assay after treatment with 1 μM doxorubicin for 48 hours in the presence or absence of IL-6 and 1 μM JAK kinase inhibitor Ruxolitinib or 20 μM STAT3 inhibitor STA-21. Cells were pre-treated with IL-6 (0.1, 1 or 10 ng/mL) for 16 hours. Cell survival is shown as percentage of viable cells compared to untreated cells i.e. in the absence of doxorubicin, IL-6 and JAK/STAT3 inhibitors (S.E.M. is indicated by bars; N=6). (**C**) p53 levels in LNCaP cells after treatment with 1 μM doxorubicin for 8 hours in the presence or absence of IL-6 and 1 μM JAK kinase inhibitor Ruxolitinib, or Pyridone 6, 1 μM STAT3 inhibitor Stattic or 20 μM STA-21. Cells were pre-treated with IL-6 (10 ng/mL) for 16 hours. The blot was cut into two pieces and probed with anti-p53 DO-1 and anti-GAPDH antibody separately.

**Figure S6. Database analysis of the IL-6/JAK/STAT3 pathway, MDM2 and TP53 in prostate cancer.** (**A**) mRNA Expression, RSEM (Batch normalized from Illumina HiSeq_RNASeqV2) of IL-6R (log 10 scale) from 32 TCGA PanCancer Atlas studies of various cancer types based on patients without any TP53 alterations and separated for no MDM2 alterations or MDM2 amplification . N = 6233 no MDM2 alterations, and N = 197 MDM2 amplification. Mann Whitney test, ***p = 0.0007.
