# Supplementary material for: Interleukin-6 derived from cancer-associated fibroblasts attenuates the p53 response to doxorubicin in prostate cancer cells
Source: Cell Death Discov. 2020 Jun 2;6:42. doi: 10.1038/s41420-020-0272-5 (PMC7265343; doi:10.1038/s41420-020-0272-5)
Supplement: Supplementary file 8 — Supplementary Table 1 [file 41420_2020_272_MOESM8_ESM.docx]

|  |  | **Prostate Adenocarcinoma^*^** | | **Metastatic Prostate Adenocarcinoma^#^** | | |
| --- | --- | --- | --- | --- | --- | --- |
|  |  | No TP53 alterations (N=410) | TP53 alterations (N=79) | | No TP53 alterations (N=256) | TP53 alterations (N=173) |
| **IL-6R amplification** | Fraction: | 2,7% | 0,0% | | 16,4% | 9,2% |
|  | N: | 11 | 0 | | 42 | 16 |
| **STAT3 amplification** | Fraction: | 0,2% | 0,0% | | 4,3% | 0,6% |
|  | N: | 1 | 0 | | 11 | 1 |
| **MDM2 amplification** | Fraction: | 1,2% | 1,3% | | 6,6% | 2,9% |
|  | N: | 5 | 1 | | 17 | 5 |

**Table S1.** Frequency of amplification of IL-6R, STAT3 or MDM2 in prostate cancer with unaltered or altered TP53.

^*^ Prostate Adenocarcinoma TCGA, PanCancer Atlas study from cBioportal (Ref. 42)

^#^ Metastatic Prostate Adenocarcinoma SU2C/PCF Dream Team, PNAS 2019 study from cBioportal (Ref. 41)
